# Influence of hemolysis, lipemia and bilirubin on biobank sample quality– origin and interference in the use for extracellular vesicle (EV) and MiRNA analyses

**DOI:** 10.1007/s00068-025-02822-w

**Published:** 2025-03-26

**Authors:** Birte Weber, Wolfgang Welz, Inna Schaible, Jiaoyan Han, Dirk Henrich, Ingo Marzi, Liudmila Leppik

**Affiliations:** Department of Trauma Surgery and Orthopedics, Goethe University Frankfurt, University Hospital, 60590 Frankfurt, Germany

**Keywords:** Exosomes, Nutrition, POCT, Hyperbilirubinemia, Propofol, Biobank

## Abstract

**Purpose:**

Pre-analytic interferences can influence the laboratory downstream measurements. We recognized hemolysis, lipemia and bilirubin in some of the serum/plasma samples of the NTF-Biobank from polytraumatized patients. Aim of the present study was to detect interferences, find reasons and describe the influence on downstream analyses.

**Methods:**

The study included serum samples of *n* = 88 polytraumatized patients admitted to a Level 1 Trauma Center in Germany at the ER & up to 10 days after trauma. Optical absorption spectra of UV-VIS (350–660 nm) were measured to detect hemolysis, lipemia and bilirubin. To find reasons for the interferences, clinical parameters like triglycerides (TAGs), nutrition, anaesthesia or transfusions were collected from patients’ record. Extracellular vesicles (EVs) were isolated by SEC from controls, lipidemic and hemolytic samples and analysed via NTA.

**Results:**

Within 10 days after trauma 31.8% of polytraumatized patients’ samples showed hemolysis, 12.5% showed increased bilirubin and 15.9% lipemia. Hemolysis occurred in samples mostly at the ER (18%) and was not associated with the number of red blood cell transfusions or the ISS. Both contaminants, hemolysis and lipemia interfered with EV/EV-miRNA measurements. EV miR-16-5p was significantly increased in patients with hemolysis. The presence of lipids further influenced the EV particle size distribution and concentration.

**Conclusion:**

The optical absorption spectra measurement is an easy tool for a robust pre-analytic sample controlling for the presence of interferences. Nutrition and anaesthesia were found to be related with lipemia in samples. Hemolysis and lipemia interfered with EV/EV-miRNA analysis. Therefore, the optical absorption spectra pre-analyses should be incorporated in the EV-biobank sampling.

**Supplementary Information:**

The online version contains supplementary material available at 10.1007/s00068-025-02822-w.

## Introduction

In the clinical laboratory setting, interference from endogenous or exogenous substances in blood samples can cause severe errors in manifold laboratory measurement. One of the leading pre-analytic causes of such interference is hemolysis [1]. Further, some authors described lipemia and bilirubin as another sources of potential errors in the interpretation of laboratory measurements [2]. Some standard clinical immunoassays were described to be influenced by hemolysis, bilirubin and lipemia: C-peptide, estradiol, serum folate, free thyroxin, insulin, and vitamin B12 were found to be affected by hemolysis; while brain natriuretic peptide (BNP), estradiol, triiodothyronine, and homocysteine were affected by high bilirubin concentrations, and BNP, serum folate, and homocysteine were affected by lipids [3]. Other authors showed further an influence of hemolysis on the coagulation tests and affected prothrombin time and partial thromboplastin time parameters [4]. In clinical settings, such interferences can pose significant challenges for interpreting measured values and may lead to serious, potentially fatal consequences for patients. Additionally, such pre-analytic interferences can also affect various results in the research settings when working with such patient samples.

As part of our routine, our research group is collecting serum and plasma samples from polytraumatized patients for the NTF-Biobank, and more recently for the newly established NTF-EV-Biobank. The nationwide biobank for serum and plasma samples from polytraumatized patients was initiated in 2013 by the task force Network Trauma Research (Netzwerk Traumaforschung, NTF) of the German Trauma Society (Deutsche Gesellschaft für Unfallchirurgie e.V., DGU) [1]. This quality-controlled project was introduced to systematically evaluate and monitor the (patho-) physiological profiles of polytraumatized patients and develop clinical strategies to improve outcomes, within a multicentre cohort. Its aim was to address the complexity of the post-traumatic immune response and identify novel research pathways by collecting biomaterials and clinical data from polytrauma patients nationwide [1]. Recently, the extracellular vesicles (EVs) were added to the biobank sampling of the NTF, because EVs play an important role as mediators and biomarker in polytraumatized patients [5–7]. Based on the new “minimal information for studies of extracellular vesicles” MISEV 2024 [8], extracellular vesicles are particles released by cells, are delimited by a bilayer and cannot replicate on their own [8].

During the sampling and processing for the biobank, visual inspection revealed that some samples exhibited signs of hemolysis, while others showed turbidity, even without the use of specialized equipment. Therfore, we decided to quantify this subjective impression by measuring the absorption spectrum of each sample of our biobank from 2023 to detect hemolysis, lipemia and bilirubin. The question arises what endogenous or exogenous factors contribute to these abnormalities, particularly in trauma patients, and how does this might influence the downstream analyses (EVs and EV-miRs) that we are currently focusing on.

## Materials and methods

### Study design

All analyses were performed with ethical approval given by the Local Ethics Committee of the University of Frankfurt (approval ID 89/19). The study was performed in accordance with the ethical standards as laid down in the Declaration of Helsinki (2013). The study includes *n* = 88 polytraumatized patients (ISS ≥ 16) admitted to the Frankfurt University Hospital Level 1 Trauma Center (Frankfurt am Main, Germany) in year 2023. The blood samples were collected at Emergency room (ER), day 1, 2, 3, 5, 7 and 10 after trauma, immediately kept on ice and serum samples were gained by 15 min of centrifugation at 3500 g and 4 °C. Blood samples from healthy controls (*n* = 4) were processed in the same way as patient’s samples.

### Clinical data

Clinical parameters such as hemoglobin (HB), bilirubin, triglycerides (TAG) values and Injury Severity Score (ISS), as well as patients’ nutrition (enteral, parenteral, whole food, vitamins or empty-stomached) and medications (especially propofol anaesthesia) were collected from the patients’ electronic record.

### Absorption measurements

50 µL of the serum were transferred to transparent, flat-bottom 96-well plates (Sarstedt, Nümbrecht, Germany) and optical absorption spectra of UV-VIS (350–660 nm, 10 nm interval) were measured by mean of a microplate reader (TECAN, Mannedorf, Switzerland).

### EV isolation and EV-miRNAs expression analyses

EVs were isolated from 100 µl randomized serum samples with detected hemolysis *n* = 4, samples with detected lipemia *n* = 4 and *n* = 4 healthy-control serum samples via Size Exclusion Chromatography (SEC) (Cell Guidance System, Cambridge, UK) according to the manufacturers protocol. miRNA was isolated from EVs via miRNeasy Serum/Plasma advanced kit (Qiagen Inc., Hilden, Germany with addition of spike-in controls UniSp 2, 4 and 5 according to the manufacturers protocol. cDNA synthesis was performed via miRCURY LNA RT Kit (Qiagen Inc., Hilden, Germany) according to the manufacturer’s instructions. RT-qPCR amplification was performed with the miRCURY SYBR Green PCR Kit (Qiagen Inc., Hilden, Germany) and commercially available primers (hsa-miR-16-5p and cel-miR-39-3p; miRCURYLNA TM miRNA PCR Assay, Qiagen Inc). Amplification was performed in CFX96 TouchReal-Time PCR Detection System (BioRad, Heidelberg, Germany) as follows: 1 cycle with 3 min at 95 °C, and 40 cycles with 10 s at 95 °C and 50 s at 56 °C. All reactions were performed in duplicates and the delta Ct method (2 ^−∆Ct^) was used for relative quantification of miR-16-5p using the Ct values of spike-in cel-miR-39-3p. miR-16-5p is described to be highly expressed in red blood cells.

### Effect of lipemia on EV particle number and size-distribution

Prior EV isolation, 20% of SmofKabiven (Fresenius kabi) were added to the serum sample from healthy control (without any sign of lipemia or hemolysis) and EVs were isolated via SEC (Cell Guidance System) according to the manufacturer’s instructions. SmofKabiven is a parenteral nutrition, which is commonly used in the ICU setting consisting of an amino acid solution with electrolytes, glucose solution and lipid emulsion. The experiment was performed in five technical replicates. EV isolates were diluted 100 times with 0.2 μm filtered PBS and EVs particles’ number and size-distribution were measured via NTA (NanosightPro, Malvern panalytical GmbH, Kassel, Germany) and data were analysed with NS Xplorer software (Malvern panalytical GmbH).

*Statistical analyses*: All statistical analyses were conducted with Graph Pad-Prism 9 (Dotmatics, San Diego, CA, USA). Data were analysed via a Kruskal–Wallis test followed by Dunn‘s multiple comparisons test. For the statistical analyses of two groups, the Mann–Whitney test was performed. Analyses with a p-value ≤ 0.05 were considered significant. Data are presented as mean ± standard error of the mean (SEM).

## Results

### Overview of samples

**Fig. 1:**
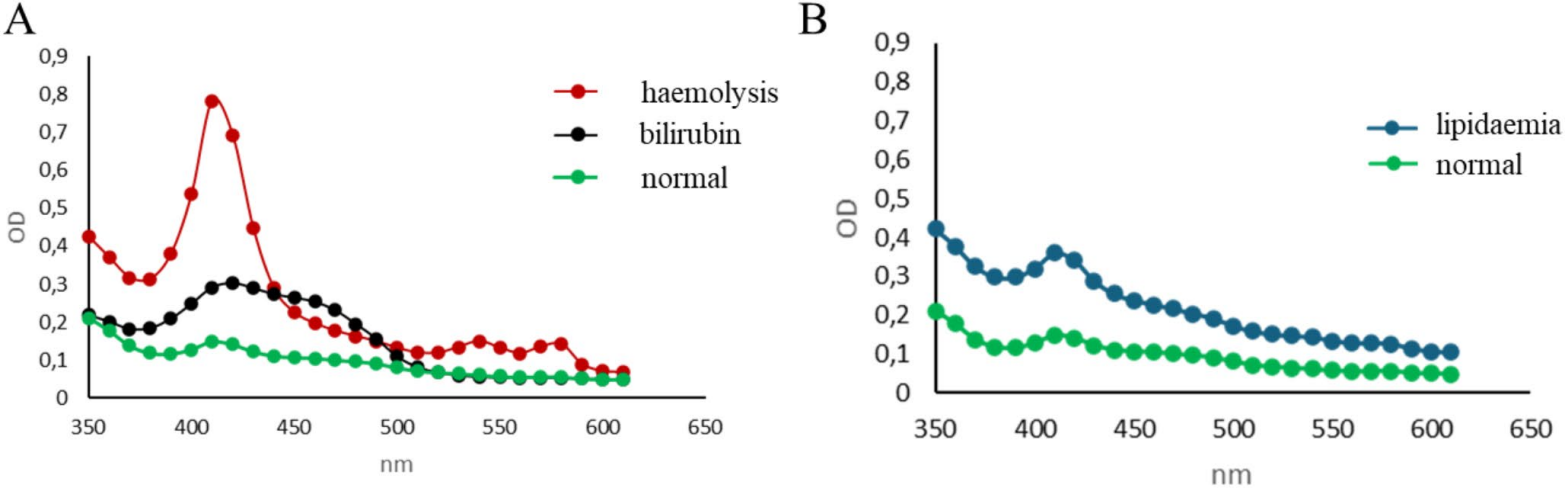
Representative polytrauma patients’ serum-absorbance spectrums. (**A**) Hemolysis (red), bilirubin (black) and normal/inconspicuous samples (green). (**B**) Absorbance spectrum of representative lipemia (blue) and normal/inconspicuous samples (green). OD = optical density

In the present study, we included 88 polytraumatized patients with an ISS ≥ 16 (Mean ISS 26.1 ± 10.0). Visual inspection of the serum samples revealed that some of the collected samples were hemolytic (red-coloured) and some of the samples looked turbid. To quantify this subjective impression, we collected the absorption spectrum of each sample (350–650 nm) and compared them. According to these measurements we subdivided the patients’ samples in hemolytic (absorbance peaks 410 nm), lipidemic (overall spectrum shift up) or bilirubin-samples (absorbance flat peak from 400 to 470 nm), illustrated in Fig. 1. We observed that 31.8% of polytraumatized patient’s samples showed hemolysis within 10 days after trauma. 12.5% of polytraumatized patients have increased levels of bilirubin in samples and 15.9% of polytraumatized patients’ samples showed lipemia within 10 days after trauma. In details, the percentage of hemolytic, lipidemic and bilirubin-samples at the different timepoints (ER up to 10 days after trauma) are presented in Table 1.

**Table 1:** Number of hemolytic, lipemic and bilirubin-containing serum samples in polytrauma patients. *n* = 88; er = emergency room

Serum samples with:	Days after trauma						
	day 0 (ER)	day 1	day 2	day 3	day 5	day 7	day 10
Hemolysis	16	4	4	4	1	2	3
Lipemia	4	5	4	3	3	3	0
Bilirubin	3	3	2	4	1	1	4

## Potential clinical cause of hemolysis and lipemia

After assessing the sample’s quality via absorbance measurement with regard to hemolysis, lipemia, and bilirubin, we aimed to determine whether the quality of the samples could be explained by polytrauma severity (ISS) and whether there is any association with routine laboratory parameters, as well as with the treatment and outcome of the trauma patients.

Interestingly, we observed that 18.1% of the trauma patients’ samples collected at ER showed hemolysis. Therefore, we analysed, if the same patients have remarkable hemoglobin levels measured in the routine laboratory. We observed that, at the ER, these patients have slightly decreased hemoglobin (Hb) levels and during the in-hospital course, it further decreased and reached a minimum at day 3 after trauma (Fig. S1A). In contrast, as shown in Figure S1B, the patients with no hemolysis in the samples showed a trend towards lower hemoglobin levels measured at the ER, which continuously and significantly decreased until day 10 after trauma Fig. S1C).

To prove if the presence of hemolysis is associated with severity of trauma and/or red blood cells transfusion, we compared ISS values (Fig. S1D) and number of transfusions (Table 2) among the groups. No significant differences among trauma severity and no association between the number of transfusions and the presence of hemolysis were detected. Most of the red blood cell transfusions were administered due to hemorrhagic shock in a single patient. The number of red blood cell transfusion are listed in Table 2. The number of red blood cells transfusions was always higher in the patients without hemolysis compared to hemolytic samples with the exception of day 3 after trauma (Table 2), where it was lower.

**Table 2:** Number of red blood cell transfusions in polytrauma patients with hemolytic and non-hemolytic samples. ER = Emergency room

Polytrauma patients with:	Days after trauma						
	day 0 (ER)	day 1	day 2	day 3	day 5	day 7	day 10
Hemolytic samples	0	2	4	2	0	0	0
Non-hemolytic samples	28	5	4	6	4	1	1
Ratio of Blood Transfusion/hemolytic samples	0	0.07	0.14	0.07	0	0	0
Ratio of Blood Transfusion/Non-hemolytic samples	0.46	0.08	0.07	0.1	0.07	0.02	0.02

**Fig. 2:**
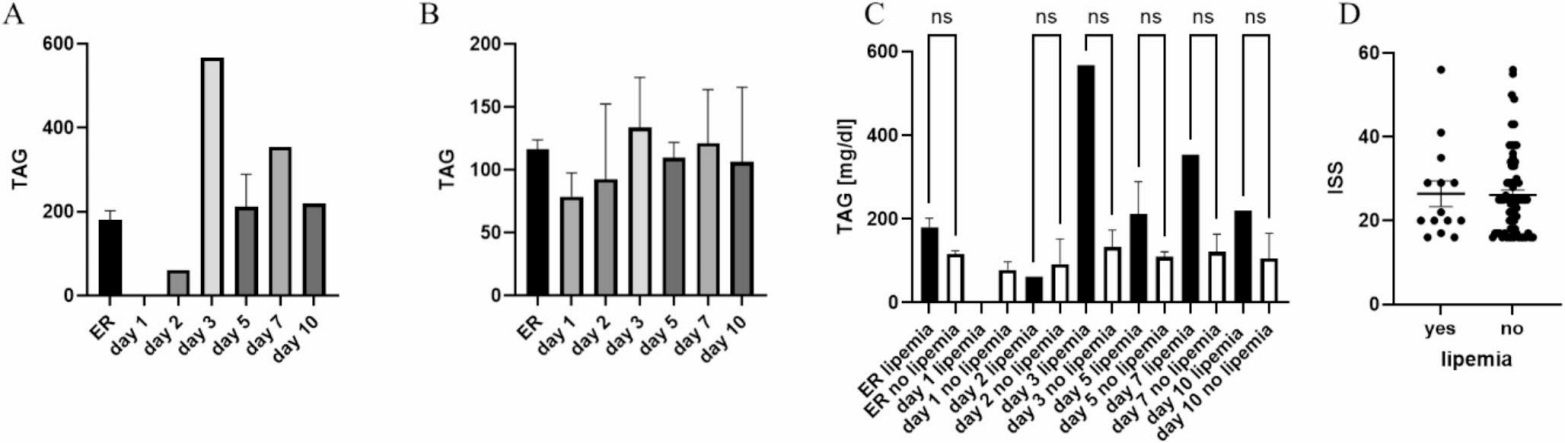
Triglyceride (TAG) concentrations in polytrauma patients with and without lipemic samples. (**A**) TAG concentration in mg/dl over 10 days after polytrauma in lipemic samples, (**B**) TAG levels in non-lipemic samples. (**C**) Comparison of TAG levels in lipemic samples compared to non-lipemic samples. (**D**) Injury severity Score (ISS) as parameter of injury severity in polytraumatized patients in regard to lipemia in biobank samples. ER = Emergency room, ISS = Injury severity Score, lipidemic group *n* = 14, non-lipidemic group *n* = 74

As described in the literature, the presence of lipemia (and therefore a turbid look) could have a significant effect on the downstream analyses of serum samples. Therefore, we investigated the appearance of lipemia in the biobank samples and tried to find the clinical reasons for its appearance. First, the possible associations of lipemia and triglyceride (TAG) concentrations in serum samples as well as trauma severity were investigated (Fig. 2). A by-trend difference in ER TAG concentrations between both groups of patients was found (Fig. 2C). Thus, the TAG concentration in patients with lipemia was twice as high as one in patients without lipemia (Fig. 2A and B). In addition, no connection among severity of trauma and presence of lipemia was found (Fig. 2D).

Next to serum TAG levels, the possible connection of iatrogenic nutrition and appearance of lipemia in serum samples was investigated. Therefore, we collected and reviewed the information of nutrition of the lipemia and the non-lipemic patients (Table 3). We observed that most of the patients in both groups were empty-stomached at the day of trauma (ER). Over the time, most of the patients in both groups received enteral tube feeding or whole food. In both groups, the portion of patients, which were fed with parenteral nutrition was quite low (Table 3).

**Table 3:** Nutrition of polytraumatized patients with lipemia (w) compared to (w/o) in percentage

	ER (w)	ER (w/o)	D11 (w)	D1 (w/o)	D2 (w)	D2 (w/o)	D3 (w)	D3(w/0)	D5 (w)	D5 (w/o)	D7 (w)	D7 (w/o)	D 10 (w)	D10 (w/o)
Fresubin		1%	15%	11%	38%	16%	33%	26%	43%	29%	42%	34%	47%	33%
Cernevit	7%		46%	41%	38%	40%	40%	28%	21%	27%	8%	22%	13%	19%
SmovKabiven central				5%		4%		7%	7%	15%	17%	9%	7%	14%
Soft food			15%	16%	6%	10%		10%		9%	8%	9%	7%	
Empty-stomached	93%	96%		8%		6%	7%	2%	7%	3%		3%		5%
Whole food		3%	23%	19%	19%	24%	20%	26%	21%	18%	25%	22%	27%	29%

ER = Emergency Room. Fresubin = enteral tube feeding (2 kcal HP fibre); Cernevit = 12-in-1 solution of essential vitamins: soluble vitamins B1, B2, B5, B6, B12, C, biotin, niacin, and folic acid, as well as lipid-soluble vitamins A, D3, and E; Smovkabiven central = parenteral nutrition; Soft food = Tea, Soup, Yoghurt, Whole food = desired food. *n* = 14 (w), *n* = 74 (w/o)

Furthermore, as anaesthesia with propofol, a whitish intravenous anaesthetic drug containing a high amount of soybean oil, might influence the turbid appearance of plasma samples, we compared the use of this drug in both groups. We observed that most of the patients in both groups received an anaesthesia with propofol on the first day after trauma (Table 4). This is also the timepoint, when the highest portion of lipidemic samples was observed. A moderate association between lipidemic samples and the use of propofol was found (*r* = 0.64).

**Table 4:** Number of patients with Propofol anaesthesia in lipemic and non-lipemic samples over 10 days after trauma. Lipemic group *n* = 14, non-lipemic group *n* = 74, er = emergency room

	ER	Day 1	Day 2	Day 3	Day 5	Day 7	Day 10
Number of patients with propofol anaesthesia in lipemic samples	1	4	1	1	1	1	1
Number of patients with propofol anaesthesia in non-lipemic samples	7	22	8	6	1	1	2
Ratio of patients with propofol/lipemic samples	0.07	0.28	0.07	0.07	0.07	0.07	0.07
Ratio of patients with propofol/no lipemic samples	0.09	0.29	0.1	0.08	0.01	0.01	0.02

Lipidemic group *n* = 14, non-lipidemic group *n* = 74, ER = Emergency room

**Fig. 3:**
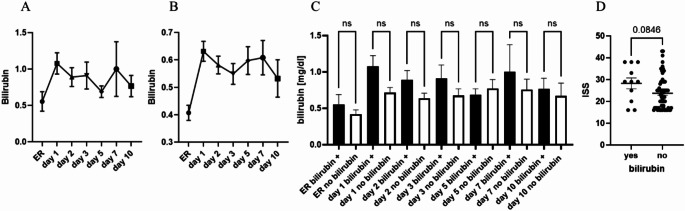
Bilirubin concentration is associated with injury severity in polytrauma patients. (**A**) Bilirubin concentration in mg/dl in bilirubin-samples. (**B**) Bilirubin levels in non-bilirubin-samples over 10 days. (**C**) Comparison of bilirubin levels of bilirubin^+^ samples compared to no bilirubin samples. (**D**) Injury Severity Score (ISS) in regard to bilirubin- or non-bilirubin-samplesER = Emergency room, bilirubin group *n* = 11, non-bilirubin group *n* = 77

The third serum parameter, which was detectable via absorbance spectrum, was serum bilirubin (Fig. 1A). Overall, 12.5% of analysed polytrauma serum samples showed bilirubin in the adsorption measurements (bilirubin^+^). We reviewed the bilirubin concentrations in the serum samples over 10 days after trauma measured in the clinical routine laboratory. In the bilirubin^+^ samples, the laboratory bilirubin concentrations were also higher compared to the no-bilirubin-samples (Fig. 3A and B), although not significant (Fig. 3C). 55% of the samples identified as bilirubin^+^ in the photometric analyses, showed bilirubin concentrations above 1 mg/dl in the routine diagnostic. The strongest increase in the bilirubin concentration was observed in the groups between ER and day 1. This is also the time slot were the Hb level decreased the most (Fig. S1). Therefore, there seemed to be an association between the bilirubin concentration and the Hb decrease. We further investigated if there is any association between the ISS as marker of the injury severity and the increase of bilirubin in the polytrauma samples. By trend, the ISS was also increased in the group of patients with increased bilirubin (Fig. 3D). Caused by this observation and the association in the literature that bilirubin could be associated with the trauma severity and outcome, we analysed the time in the hospital, the time on ICU or IMC and the survival of patients with increased bilirubin in samples compared to the patients without bilirubin increase. By-trend the time in hospital and the time on ICU/IMC was increased in bilirubin^+^ patients (Data not shown). Non-survival was higher in the bilirubin^+^ group compared to the no bilirubin group (18.2% vs. 15.6%) (Data not shown). Interestingly, in case of one patient with a hemorrhagic shock, all serum samples collected over 10 days after trauma were positive for bilirubin via photometric analyses.

### Possible negative effects of hemolysis/lipemia on downstream applications (EV research)

Next to the possible clinical cause of hemolysis and lipemia in serum samples, the potential negative effects of these contaminants on downstream applications (EV research) was evaluated.

**Fig. 4:**
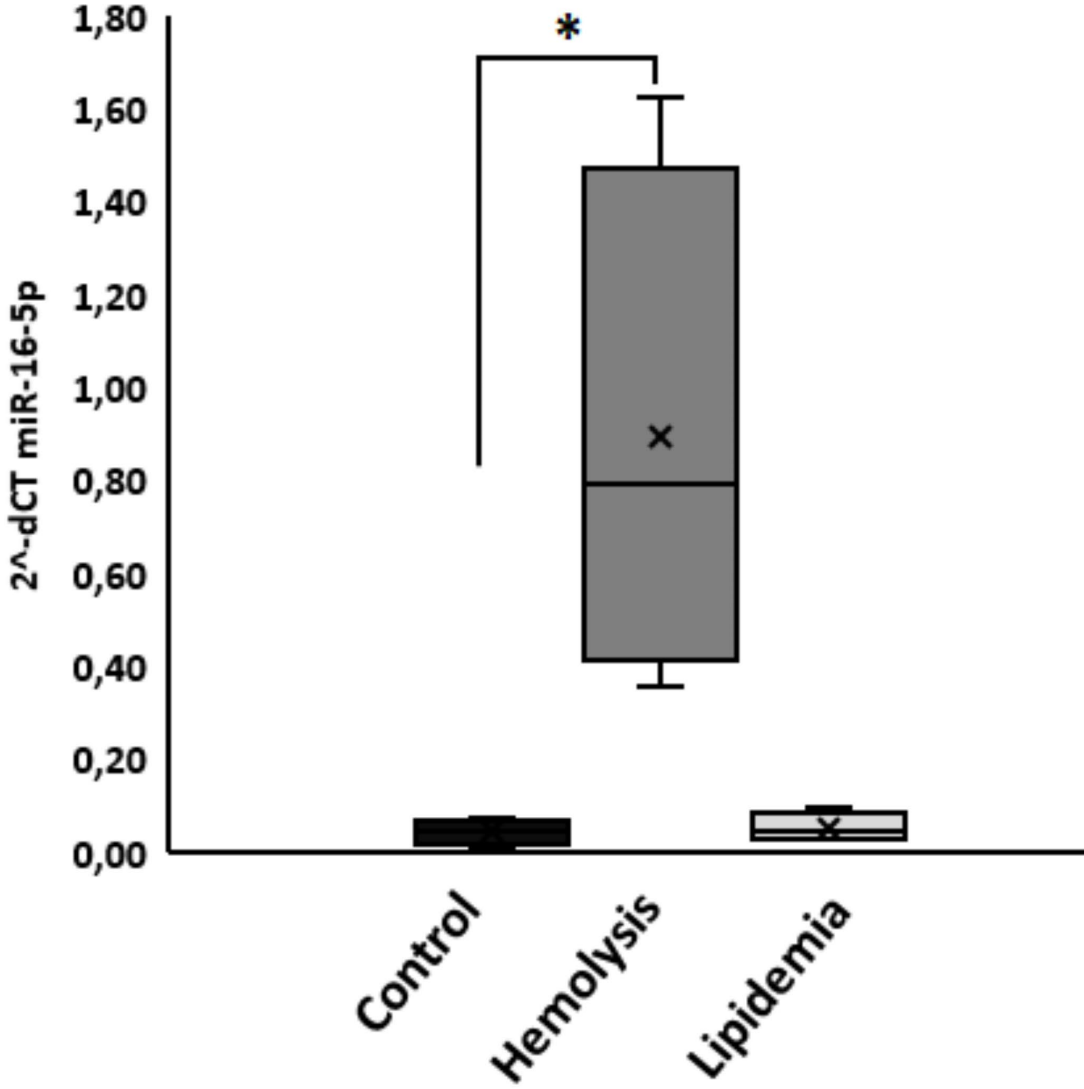
Hemolysis in polytrauma patients is accompanied by EV-miR-16-5p contamination. Measurement of miR-16-5p expression by qPCR in hemolytic, lipemic and control samples after EV isolation via size exclusion chromatography (SEC). *n* = 4 individual patients in each group, *p ≤ 0.05

Because hemolysis or the presence of damaged red blood cells, in serum samples could affect among others EV-miRNA expression analyses, we measured the concentration of EV miR-16-5p in polytrauma samples with hemolysis, lipemia and inconspicuous samples. A significant increase of miR-16-5p in EVs of polytrauma patients with hemolysis but not with lipemia or control samples was found (Fig. 4).

**Fig. 5:**
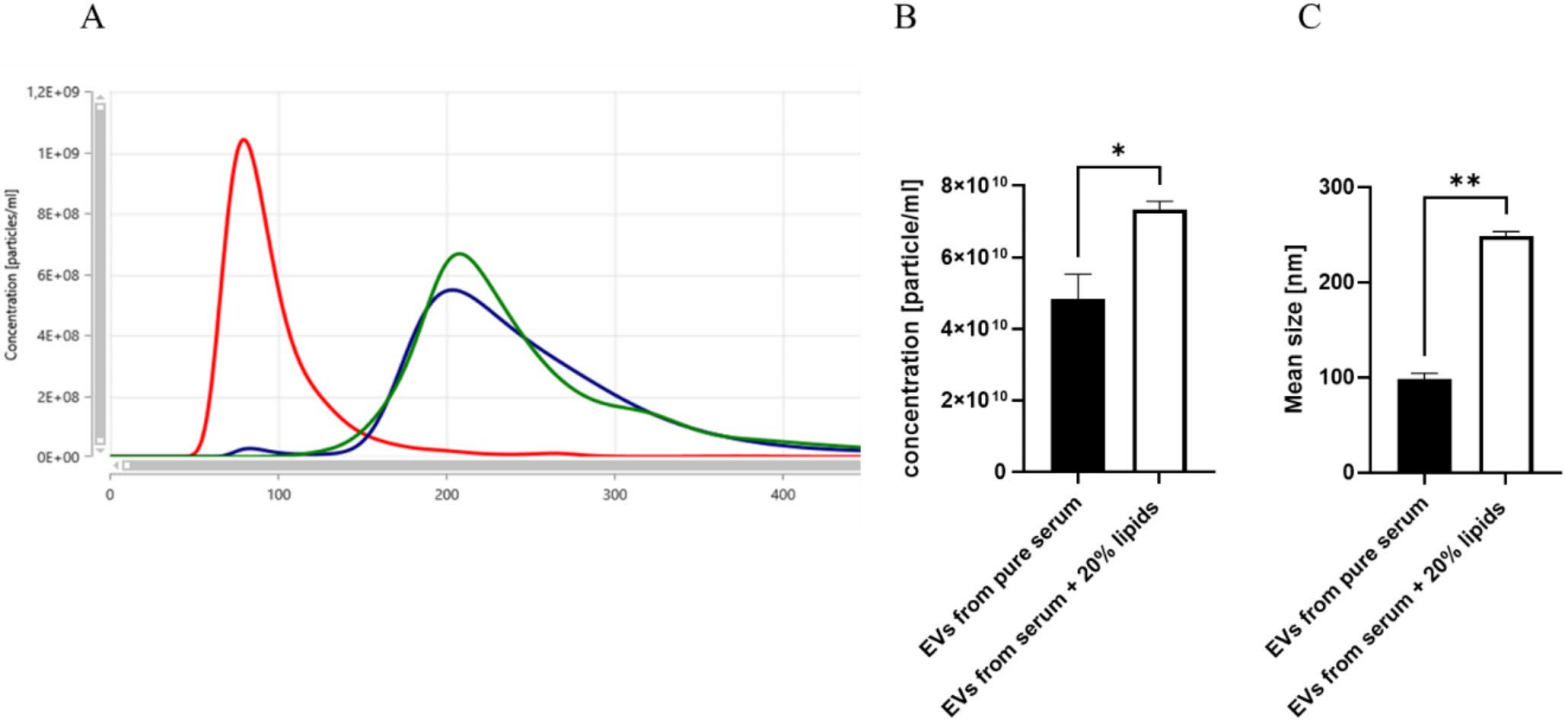
Serum contamination with lipid-reach nutrition solution SmofKabiven influenced nanoparticle tracking analysis (NTA) of EV samples. (**A**) Representative NTA of control serum EVs (in red), compared with two representative EV samples isolated from serum with 20% SmofKabiven (blue and green). (**B**) Particle concentration measured by NTA in EVs isolates from pure serum (healthy volunteer) in comparison to EVs isolates from serum (same healthy volunteers) + 20% SmofKabiven. (**C**) Particle size analyses of EVs from control serum samples compared to EVs from artificially modified serum samples. *n* = 5, *p ≤ 0

As spheric lipoprotein particles are known to overlap by size and form with EVs, we evaluated the possible interference of lipemia and EV particle number and size distribution analyses via NTA. In order to do so and mimic lipemia samples, the SmofKabiven central solution (whitish, fat-rich parenteral nutrition) was first added in different concentrations (0%, 0.2%, 1%, 20%, 40%, 60%, 80% and 90%) to the control serum samples. The analyses of adsorption spectrum of these serum samples showed that rising percentage of SmofKabiven in the samples shift up the absorption curve in the same way as it happened in samples with lipemia (data not shown). Moreover, the samples with ≥ 20% SmofKabiven contamination were found to be comparable with real clinic lipemic samples by means of absorbance spectrum and were chosen for further analyses. Hypothesising that lipid contamination of serum could influence EV analyses via NTA we performed control experiments, in which EVs were isolated from control serum samples (without lipids contamination) and from the same serum samples contaminated with 20% SmofKabiven. The results showed a change in the EV-size distribution detected by NTA, as demonstrated in Fig. 5A. The pure serum sample was used to isolate EVs (red), which demonstrated a mean size of 80 nm, while the artificially modified samples with 20% SmofKabiven showed EVs with a mean size of 200 nm (green and blue in Fig. 5A). As demonstrated in Fig. 5C, this difference in size was significant. Further the NTA detected particle concentration was also influenced by the presence of lipids in samples. In Fig. 5B, the mean concentration of pure serum EVs is significantly lower compared to the concentration of EVs isolated from artificially modified with + 20% SmofKabiven serum (Fig. 5B).

## Discussion

Biobanking of patient samples plays a critical role in advancing medical research, enabling the development of personalized therapies and improving diagnostic tools. However, obtaining blood samples from polytrauma patients, particularly during emergency room visits, can be challenging due to the urgency of treatment and potential difficulty in accessing veins. As part of a biobank managing routine sample collection, we have become increasingly aware of visual differences in samples, highlighting the importance of careful monitoring for potential contaminants that may affect downstream analyses. The goal of this study was to visually inspect routinely collected serum biobank samples and analyse them using spectrophotometry for hemolysis, lipemia, and bilirubin contaminants. The focus was to identify contaminated samples and determine if these contaminants could be linked to patient factors. Additionally, the study aimed to assess whether contaminants affect the use of these samples in downstream analyses, like EV and EV-miRNA studies.

For serum sample quality control, we chose optical absorption analysis, a fast method that does not require complex equipment or specialized personnel. It has been previously used to detect hemolysis, icterus (bilirubin), and lipemia interference in serum and plasma samples and several modern biochemistry analysers have been developed for clinical use [9].

### Hemolysis

Hemolysis is well known the most common pre-analytical error affecting biochemical analyses of liquid biopsy samples. Hemolysis was the most common quality issue, occurring in 31.8% of our patients’ samples. Most hemolytic samples were from the ER, consistent with literature reporting that 31% of ER samples are hemolytic [10]. In trauma patients several exogenous factors, like transfusion via an intraosseous administration [11], transfusion of uncross-matched red blood cell units [12], the use of veno-venous extracorporeal membrane oxygenation are discussed to be associated with the development of hemolysis [13]. Additionally trauma itself could lead to the hemolysis in these patients for example due to damaged pulmonary epithelium after severe chest trauma [14]. The literature shows that more critically injured patients, non-survivors, and those with longer ventilator use have higher hemolysis levels, with these markers also linked to increased red blood cell transfusions [15]. We did not find any association of hemolysis with the ISS, with the amount of transfused red blood cells or with the changes in hemoglobin levels in our trauma patients. In addition to pathophysiological changes, factors related to blood sample collection and processing can also contribute to hemolysis [16, 17]. Although the sampling, transport, and processing steps in our serum/plasma NTF biobank are standardized and performed by trained, experienced personnel to prevent in vitro hemolysis [18], it cannot be entirely excluded due to the complexities of sample collection from trauma patients. These complexities arise from factors such as the frequent use of intravenous catheters, prolonged tourniquet times during blood withdrawal in the emergency room, and circulatory depression in trauma patients.

Hemolysis is known to affect laboratory testing by releasing intracellular from damaged erythrocytes [19]. With the focus on the EV biobank we aimed to determine if hemolysis in serum samples also impacts EV (EV-miRNA) analyses. We compared miR-16-5p expression in EVs isolated from hemolytic, lipemic and non-affected serum samples. This miRNA was chosen due to its high expression in red blood cells and its plasma/serum concentration being influenced by RBCs contamination [20, 21]. Our findings demonstrated, that miR-16-5p was significantly increased in EVs from hemolytic samples (Fig. 4) compared to lipemic and control samples, suggesting hemolysis affects both serum miRNA and EVs miRNA measurements. These findings indicate that miRNA-16-5p should not be used as a normalization miRNA in plasma/serum studies [22, 23].

### Lipemia

Lipemia can interfere with serum/plasma biochemical analyses by altering light scattering, uneven distribution of analytes between aqueous and lipid phases, or interacting with assay reagents [9, 24, 25]. Lipemia was detected in one or more samples collected during 10 days after trauma in 15.9% of our patients. The most common cause of lipemia is non-fasting sample collection, which is unavoidable in polytrauma patients in the ER [2] or those receiving lipid-rich parenteral nutrition [26]. In our patient cohort, no link between parenteral nutrition and lipemia was found. However, this should be interpreted with caution due to the small number of patients on parenteral nutrition, the high number of fasted patients, and the misalignment between blood sample collection and the nutrition schedule. Lipemia can also result from medications like propofol, commonly used in trauma patients. Propofol-associated hypertriglyceridemia is frequent in mechanically ventilated ICU patients [27], and it has been linked to life-threatening propofol infusion syndrome in about 4% of trauma patients at level 1 trauma centers [28]. In the present patient collective, most received propofol anaesthesia on the first day after trauma, which coincide with the highest percentage of lipemic samples. This suggests a potential association between propofol use and the appearance of the turbid lipemic samples, warranting further investigation. Additionally, comparing TAG levels between lipemic and non-lipemic polytrauma samples revealed that TAG concentration at ER was almost two-fold higher in the lipemic group. The literature underscores the significance of circulating lipid analytes as prognostic markers in polytrauma patients [29, 30]. Our findings of a significant presence of lipemic samples in the biobank, potentially linked to propofol or parental nutrition, emphasize the need for awareness of this contaminant in studies focusing on circulating lipids analytes.

Thought that lipoprotein particles (similar by physical characteristics with EVs) could interfere with EV measurements in lipemic samples we compared EV isolates from control serum samples and serum samples contaminated with parenteral nutrition SmofKabiven. Our results showed nutrition contaminants significantly affected NTA measurements, with both mean particle size and concentration being significantly higher in the contaminated group. These results align with previous studies, showing that low-density lipoproteins mimic plasma-derived exosomes and that current EV-isolation methods cannot fully remove lipoprotein particles [31]. This means that lipemic samples should be avoided in studies involving EV particle-size and/or particle-concentration analyses [32].

### Bilirubin

The third main topic of the present analysis was the biobank serum samples with increased bilirubin concentration. Hyperbilirubinemia or icterus has been frequently reported in severely injured patients and was associated with poor outcome [33]. The trend observed in our study linking injury severity with increased bilirubin concentration is consistent with the literature [33]. Bilirubin, a catabolic product of hemoglobin [34], hemoglobin levels were compared between patients with and without elevated bilirubin, but no difference was found. However, in one patient with severe hemorrhagic shock and the need for > 20 RBC units, a significant increase in bilirubin was observed in all collected samples over 10 days. Interestingly, we observed the greatest increase in bilirubin concentrations between the ER and day one in both groups of patients, coinciding with the maximum hemoglobin decrease. A decrease in hemoglobin and increase in billirubin have been linked to bleeding, subarachnoid hemorrhage [35] and higher mortality in traumatic brain injury patients [34]. Patients with extreme hyperbilirubinemia (above 12 mg/dl), caused by infection, sepsis, or hypoxic hepatitis have shown high mortality rates (up to 76%) [36].

The presence of bilirubin in serum samples itself is not expected to directly interfere with EV measurements and in the current literature, is not associated with changes in the EV-miR profile, so far.

Overall, our results reveal a relatively high percentage of hemolysis, lipemia, and bilirubin-contaminated samples in our biobank, highlighting the importance of pre-analytical testing of serum samples to ensure the accuracy of downstream analyses. We recommend screening biobank samples before inclusion in research studies using simple adsorption spectrum measurements. A suggested algorithm for evaluating the spectrophotometric results is provided in supplementary Figure S2. Nevertheless, excluding biobank samples that are positive for hemolysis and/or lipemia could introduce a bias into the sample cohort, especially given the high percentage of such samples. Considering the limited number of patient samples and the potential bias introduced by excluding contaminated samples, an alternative approach could involve incorporating additional steps in the study design that would allow these samples to be retained without compromising the analysis. For example, for lipemic samples and EV particle NTA analysis, the ratio of lipemic non-EV particles could be estimated using flow cytometry with fluorescently labelled EV-epitope antibodies and/or fluorescent labelling of DNA/RNA in the EV cargo. Our findings and the approach we employed are not limited to trauma patient samples and could be extended to other patient biobanks. Furthermore, they underscore the lack of homogeneity in biobank samples, raising important questions about the feasibility of extrapolating data obtained from a representative cohort to the entire biobank.

This study has several limitations. While we focused on hemolysis, lipemia, and bilirubin, other contaminants, such as protein impurities, may also be present. Additionally, we analyzed only one miRNA, though other miRNAs could also impact EV results. Future research should thoroughly investigate the causes of contamination in biobank samples, as well as their effects on EVs, miRNAs, and other analytes measured in biobanks.

## Conclusion

Taken together, the present analysis showed that the optical absorption spectra measurement is a simple, reliable tool for quick and robust pre-analytic control of biobank samples to detect hemolysis, lipemia and elevated bilirubin levels. The high percentage of hemolytic samples in our biobank aligns with existing literature and was not associated with the number of red blood cell transfusions or trauma severity. Nutrition and anaesthesia were associated with lipemia in biobank samples. Since both hemolysis and lipemia interfere with EV/EV-miRNA measurements, incorporating optical absorption spectra analysis into routine EV biobank sample collection is recommended. This will enable informed decisions about excluding contaminated samples or, when exclusion is not possible, adjusting the study design to control contaminant levels.

## Electronic supplementary material

Below is the link to the electronic supplementary material.

Supplementary Material 1

## Data Availability

All data supporting the findings of this study are available within the paper and its Supplementary Information.
