# Supplementary material for: Influence of hemolysis, lipemia and bilirubin on biobank sample quality– origin and interference in the use for extracellular vesicle (EV) and MiRNA analyses
Source: Eur J Trauma Emerg Surg. 2025 Mar 26;51(1):153. doi: 10.1007/s00068-025-02822-w (PMC11947011; doi:10.1007/s00068-025-02822-w)
Supplement: Supplementary file 1 — Supplementary Material 1 [file 68_2025_2822_MOESM1_ESM.docx]

Supplemental Figure


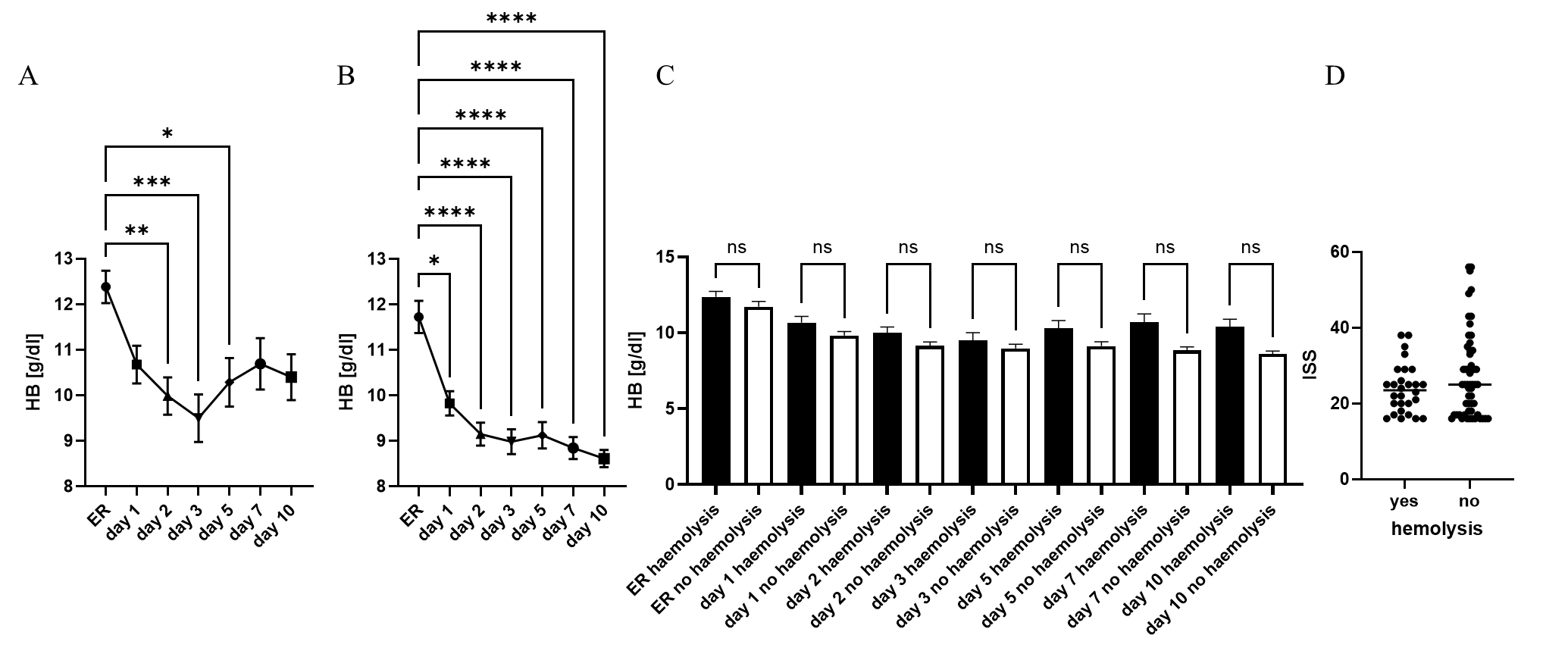


**S1** Hemolysis in polytraumatized patients’ serum samples is not associated with hemoglobin concentrations and severity of trauma. A) Hemoglobin (HB) concentrations in patients with hemolytic samples over 10 days. B) Hemoglobin concentrations in patients with non-hemolytic samples over 10 days. C) Comparison of HB levels between hemolytic and non-hemolytic patients over 10 days. D) Injury severity score (ISS) in dependency of hemolysis in samples of polytrauma patients. Hemolytic samples n = 28, non-hemolytic samples n = 60, *p ≤ 0.05, **p ≤ 0.01, ***p ≤ 0.001, ****p ≤ 0.0001

**
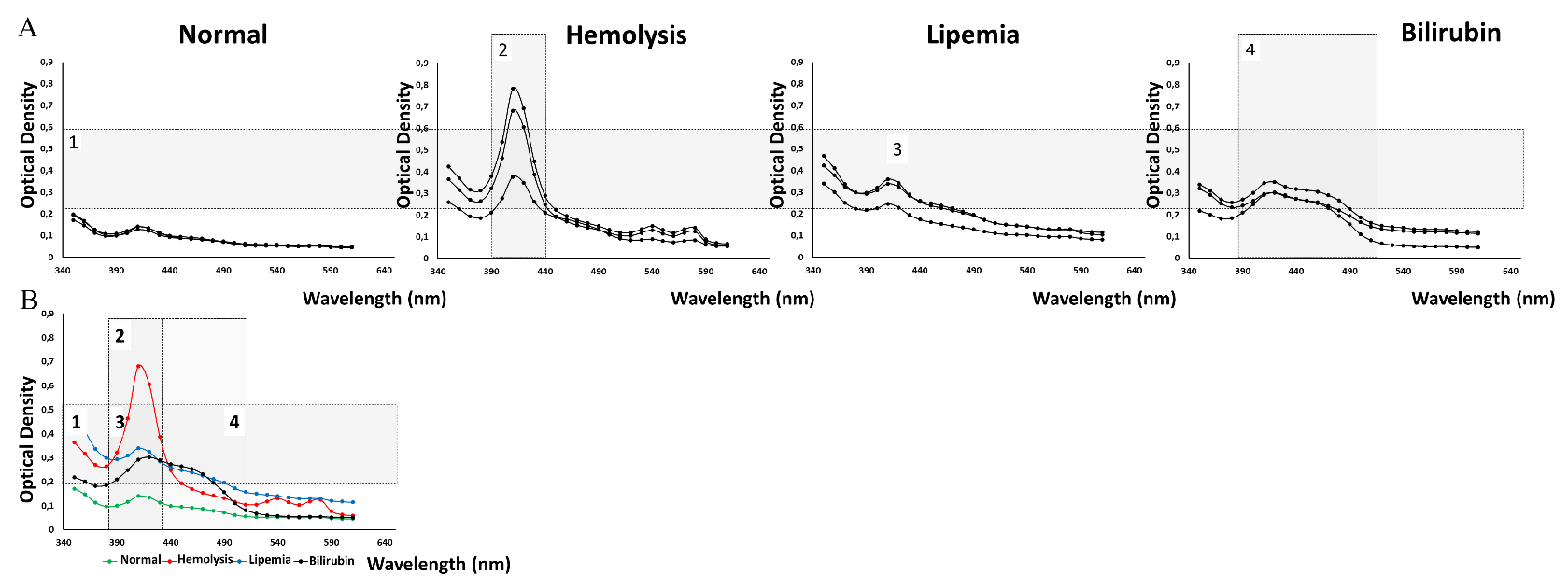
**

**S2** Evaluation of the quality of serum/plasma samples via spectrophotometry.

**A** The algorithm for evaluating the results of spectrophotometric quality control of plasma/serum samples is presented, along with representative samples (n=3) both with and without contaminants. 1) Samples which do not have OD>0.2 at any analysed wavelength (340-650 nm) do not have contaminant. 2) In case there are OD values > 0.2, special attention should be given to the region 350-450 nm wavelength. If there is a clear sharp peak of absorbance in this nm interval, samples could have hemolysis. 3) If there is no (minimal) peak of absorbance in this interval, samples are likely lipemic. 4) If there is a broad peak at 350-500 nm, samples could have increased bilirubin.

**B** In this overview of the different sample interferences, the unsuspicious curve of normal serum samples is presented in green, the hemolytic sample curve is in red, the lipemic sample curve is in blue and the bilirubin curve is in black.
